# Signs, symptoms, and treatment patterns across serial ambulatory cardiology visits in patients with heart failure: insights from the NCDR PINNACLE® registry

**DOI:** 10.1186/s12872-018-0808-2

**Published:** 2018-05-03

**Authors:** Larry A. Allen, Fengming Tang, Philip Jones, Tracie Breeding, Angelo Ponirakis, Stuart J. Turner

**Affiliations:** 1 Division of Cardiology, University of Colorado School of Medicine, Anschutz Medical Campus, 12631 E. 17th Avenue, Academic Office 1, #7019, Mail Stop B130, Aurora, CO 80045 USA; 2 Saint Luke’s Mid America Heart Institute, 4401 Wornall Road, Kansas City, MO 64111 USA; 3 American College of Cardiology, Heart House, 2400 N Street NW, Washington, DC 20037 USA; 4 Health Economics & Outcomes Research, Novartis Pharmaceuticals Corporation, One Health Plaza 125/3410B, East Hanover, NJ 07936-1080 USA

**Keywords:** Ambulatory care, Drug therapy, Heart failure, Registries, Dyspnea

## Abstract

**Background:**

Due to a relative lack of outpatient heart failure (HF) clinical registries, we aimed to describe symptoms, signs, and medication treatment among ambulatory patients with heart failure (HF) over time.

**Methods:**

Using health records from 234 PINNACLE (Practice Innovation and Clinical Excellence) U.S. cardiology practices (2008–2014), serial visits for patients with HF were characterized. Symptoms, signs, and HF medications (angiotensin-converting enzyme inhibitors [ACEI], angiotensin receptor blockers [ARB], beta blockers [BB], and diuretics) were compared between visits.

**Results:**

Among 763,331 patients with HF, 550,581 had ≥2 clinic visits < 1 year apart, with 2,998,444 visit pairs. In the 12 months following an index visit, patients had a mean of 2.5 ± 2.3 additional visits. Recorded index visit symptoms ranged from dyspnea (53.6%) to orthopnea (23.1%); signs ranged from peripheral edema (52.2%) to hepatomegaly (0.6%). Of those with ejection fraction < 40%, ACEI was prescribed in 58.6%, ARB in 18.5%, BB in 85.2%, and diuretics in 70.0%. Between-visit recorded changes were infrequent: dyspnea appeared in 3.8%, resolved in 2.7%; NYHA class increased in 2.9%, decreased in 2.9%; number of signs increased in 6.0%, decreased in 5.1%; ACEI/ARB or BB added in 6.4%, removed in 6.2%; diuretic added in 3.7%, removed in 3.8%. Changes in recorded symptoms were rarely associated with initiation or discontinuation in HF medication classes.

**Conclusions:**

Ambulatory HF care in U.S. cardiology practices seldom recorded changes in symptoms, signs, and medication class. Although templated medical records and absence of medication dosing likely underestimated the degree to which clinical changes occur over serial visits for HF, these PINNACLE data suggest opportunities for greater symptom-based and therapy-focused visits.

## Background

The nearly 6 million Americans with heart failure (HF) account for more than 12 million physician office visits each year [1–4]. From patient and clinician perspectives, these medical encounters provide an opportunity to improve the medical management of HF, which in turn has the potential to relieve the symptoms of HF, improve health-related quality of life, reduce hospitalizations, and prolong survival [5–9]. Current clinical practice guidelines recommend the serial optimization of HF medication therapies, involving dose titration and monitoring to achieve maximum symptom relief and medication tolerability [5, 7, 8, 10].

A number of patient registries have been established to characterize patients with HF and the care received in routine clinical practice. However, due to a variety of logistical issues, they have been primarily limited to in-hospital care, administrative billing data, or a small number of sites, and thus have been unable to provide a comprehensive look at ambulatory HF care across the U.S. [11–14]. The American College of Cardiology’s National Cardiovascular Data Registry’s (NCDR®) PINNACLE (Practice Innovation and Clinical Excellence) Registry® is now the largest contemporary clinical database of ambulatory cardiology care, which includes detailed information on symptoms, signs, medication prescribing, procedures, and outcomes from patients diagnosed with a variety of cardiovascular (CV) conditions [15–19]. Using this emerging real-world ambulatory view provided by the PINNACLE Registry, we sought to characterize changes in recorded symptoms, physical signs, and prescribing of medication classes across serial ambulatory cardiology visits for patients with HF.

## Methods

### Study design

PINNACLE is the first U.S. national, prospective, office-based, quality improvement registry for CV ambulatory care in the U.S. Participation is voluntary, and data are routinely collected and submitted to the American College of Cardiology’s NCDR from participating practices using paper-based chart abstraction or a mapping algorithm from electronic health records [18]. The current study was a cross-sectional analysis of patients ≥18 years of age enrolled in the PINNACLE Registry with a diagnosis code for HF between May 1, 2008, and December 30, 2014. Included patients had at least 1 pair of ambulatory visits less than 1 year apart. Diagnosis of HF was defined using PINNACLE Registry criteria of unusual dyspnea on light exertion, recurrent dyspnea occurring in the supine position, fluid retention, and low cardiac output secondary to cardiac dysfunction; via the description of rales, jugular venous distension, or pulmonary edema constituted HF; or as a previous hospital admission with primary diagnosis of HF [20, 21]. The first visit in the first pair of ambulatory visits was defined as the index visit. Baseline characteristics were assessed at the time of the first visit (index visit). To assess changes in symptoms, signs, and treatment, all subsequent visits following the index visit were compared against the visit immediately prior if the 2 visits were less than 1 year apart. Data were extracted directly from the practices’ electronic health record or via a paper-based reporting form. Data collection was standardized using established definitions, uniform data entry and transmission, and quality checks. In addition, rigorous back-end data quality checks were performed on the extracted data. Any data not meeting predefined quality thresholds were quarantined from analyses and flagged for manual review and follow-up with individual practices [18]. Missing data for any visit were handled using a last observation carried forward approach, where available.

### Study outcomes

The primary outcomes of interest were the associations between changes in the incidence of the signs and symptoms of HF and changes in medications at a class level. The signs and symptoms of HF assessed included changes in New York Heart Association (NYHA) functional class, the presence of dyspnea and orthopnea, as well as changes in the number of physical signs of HF present, including rales, ascites, peripheral edema, hepatomegaly, third heart sound (S_3_ gallop), fourth heart sound (S_4_ gallop), and jugular vein distention. Medications were classified as angiotensin-converting enzyme inhibitors (ACEI), angiotensin receptor blockers (ARB), beta blockers (BB), and diuretics.

Due to limitations in the granularity of medication capture, loop diuretics, thiazides, diuretics, and mineralocorticoid receptor antagonists were grouped as a single diuretics category during PINNACLE data collection. Therefore, MRA (spironolactone and eplerenone) cannot be distinguished from other diuretics, including commonly used loop diuretics (e.g. furosemide). Thus, “diuretics” represents a heterogeneous class in this analysis. At each visit, patients were assessed for presence or absence of a prescription for each of the 3 classes: 1) ACEI/ARB, 2) BB, and 3) diuretics. Medication treatment change between visits was classified into 1 of 3 groups: ‘increase’ (no to yes), ‘decrease’ (yes to no), or ‘no change’ in the number of HF medication classes prescribed. Currently, PINNACLE data capture does not allow for an analysis of HF medication dosing.

### Data analyses

Demographics and clinical characteristics were represented as means and standard deviations (SD) for continuous variables and frequencies and percentages for categorical variables. The association between treatment patterns and change in HF symptoms and physical assessments was described using percentages of treatment ‘increase’, ‘decrease’, and ‘no change’ in HF symptoms and physical assessment categories. Rate ratios for treatment increase and treatment decrease were also calculated for patients with HF symptoms and physical assessment increase and decrease relative to those with no change. The frequency of HF treatment modifications in 1 year of follow-up after index, number of subsequent visits after index per patient, and time between 2 consecutive eligible visits using mean, interquartile range, and SD were also described. All analyses were then repeated and stratified by age group (< 65 and ≥ 65 years), and within the subset with reduced left-ventricular ejection fraction (LVEF < 40%).

A number of key variables in PINNACLE had non-trivial missing rates. To assess for potential bias, the demographics and clinical characteristics of the visits included versus those of the visits excluded due to missing data were described using means (SD) for continuous variables and frequencies (percentages) for categorical variables. In the sensitivity analysis to evaluate the impact of potential bias due to missing values, we repeated the primary analysis using inverse probability weighting, which assigned higher weights to visits that were similar to those with missing values. The probability weights were calculated from a logistic regression model on the basis of patient demographics and clinical characteristics. Data collection was standardized through the use of standard definitions, uniform data entry and transmission requirements, and data quality checks.

## Results

### Frequency of cardiology visits among patients with HF

A total of 4,713,004 patients were identified in the PINNACLE Registry from 234 practices from May 1, 2008 to December 30, 2014. From 763,331 patients who had a documented diagnosis of HF, 550,581 patients (72.1%) from 224 practices had at least 2 ambulatory visits less than 1 year apart. The total number of qualified visit pairs was 2,998,444. The mean length of follow-up per patient was 1.9 ± 1.6 years. In the 12 months following an index visit, patients had a mean of 2.5 ± 2.3 additional visits.

### Findings at index visit

The baseline demographics and clinical characteristics of the study population are presented in Table 1. The mean (SD) age of the population was 69.7 (13.4) years, 45.1% were women, and 87.4% were white. LVEF was available in 52.2% of the patients; 26.1% of patients with an LVEF measure had a LVEF < 40% at the index visit. The most common comorbidities were hypertension (82.2%) followed by dyslipidemia (65.0%) and coronary artery disease (60.9%).

**Table 1:** Baseline patient demographics and clinical characteristics

	OVERALL STUDY POPULATION (*n* = 550,581)	Patients with Data Available	Patients with LVEF < 40% (*n* = 75,107)	Patients > 65 Years of Age (*n* = 374,580)
Age, yrs	69.7 ± 13.4	100%	68.6 ± 13.1	77.0 ± 7.6
Sex		99.7%		
Men	54.9%		69.1%	53.2%
Race		42.5%		
White	87.4%		86.3%	91.1%
Black	10.1%		12.0%	6.7%
Other	2.5%		1.7%	2.2%
Ethnicity				
Hispanic	1.9%	93.7%	2.2%	1.8%
Comorbidities				
Hypertension	82.2%	92.5%	77.1%	84.7%
Dyslipidemia	65.0%	86.9%	68.6%	68.4%
Coronary artery disease	60.9%	93.9%	70.7%	65.3%
Atrial fibrillation/flutter	34.2%	92.0%	35.8%	41.2%
Type 2 diabetes	28.1%	94.4%	30.7%	28.2%
Previous myocardial infarction	21.7%	86.8%	31.4%	22.4%
Stable angina	11.5%	86.7%	11.2%	12.5%
Peripheral arterial disease	10.8%	87.9%	11.4%	12.4%
Stroke/transient ischemic attack	6.1%	77.6%	5.8%	7.1%
Ischemic stroke	2.5%	36.5%	2.5%	2.9%
Unstable angina	2.4%	96.1%	2.4%	2.5%
NYHA functional class		32.0%		
1	59.7%		29.0%	54.0%
2	29.3%		46.5%	33.1%
3	10.0%		22.3%	11.8%
4	1.0%		2.3%	1.1%
Symptoms				
Dyspnea	53.6%	85.7%	57.4%	55.2%
Orthopnea	23.1%	88.7%	24.5%	23.3%
Systolic BP, mm Hg	127.7 ± 19.5	93.0%	121.8 ± 19.0	128.1 ± 19.4
Diastolic BP, mm Hg	73.2 ± 11.6	93.0%	71.7 ± 11.8	71.3 ± 10.9
Heart rate, bpm	72.9 ± 13.6	86.6%	74.1 ± 13.6	72.0 ± 13.2
Physical signs				
Rales	4.9%	86.2%	6.2%	5.7%
Ascites	0.8%	92.9%	1.1%	0.8%
Peripheral edema	52.2%	77.2%	54.2%	53.4%
Hepatomegaly	0.6%	96.1%	1.0%	0.6%
S_3_ gallop	6.9%	91.8%	8.6%	7.3%
S_4_ gallop	12.3%	89.1%	11.6%	12.8%
JVD	6.8%	91.8%	8.4%	7.2%
BMI, kg/m^2^	30.8 ± 9.6	74.3%	29.9 ± 9.0	29.6 ± 9.0
Tobacco use		52.1%		
Never	40.1%		34.3%	40.0%
Current	14.4%		17.0%	11.2%
Quit within 12 months	3.3%		4.0%	3.2%
Quit more than 12 months ago	42.1%		44.7%	45.7%
LVEF		52.2%		
≥50%	58.3%			60.5%
40–49%	15.6%			15.5%
< 40%	26.1%		100%	24.0%
Treatment		100%		
ACEI	43.6%		58.6%	42.3%
ARB	20.7%		18.5%	22.1%
BB	68.8%		85.2%	70.0%
Diuretic	56.4%		70.0%	60.0%
Medical procedures/ devices				
PCI	1.0%	70.1%	1.3%	1.0%
Pacemaker	9.2%	35.2%	24.1%	10.1%
CRT-D	9.5%	34.9%	24.4%	10.4%
ICD	11.1%	34.8%	29.7%	11.5%

*ACEI* angiotensin-converting enzyme inhibitor, *ARB* angiotensin receptor blocker, *BB* beta blocker, *BMI* body mass index, *BP* blood pressure, *bpm* beats per minute, *CRT-D* cardiac resynchronization therapy plus defibrillator, *ICD* implantable cardioverter defibrillators, *JVD* jugular vein distention, *LVEF* left ventricular ejection fraction, *mm Hg* millimeters of mercury, *NYHA* New York Heart Association, *PCI* percutaneous coronary intervention, *S*_3_ third heart sound, *S*_4_ fourth heart sound, *yrs*. years

Symptoms of dyspnea and orthopnea were reported in 53.6% and 23.1% of patients at the index visit, respectively. Patients were predominantly in NYHA functional class 1 (59.7%) at the index visit, with an additional 29.3% of patients in class 2. The most frequently reported physical sign of HF was peripheral edema (52.2%), followed by S_4_ gallop (12.3%). At the index visit, an ACEI/ARB or BB were not prescribed for 17.5% of patients; 68.8% were prescribed BB, 43.6% were prescribed ACEI, and 20.7% were prescribed ARB; 47.3% of patients were on a combination of an ACEI/ARB plus a BB and 35.1% were on either an ACEI/ARB or a BB. Diuretics were prescribed for 56.4% of patients.

A number of variables in PINNACLE had a significant proportion of missing data across visits: NYHA (66.1% missing), dyspnea (17.1% missing), orthopnea (11.4% missing), and any of the 7 physical signs, including rales, peripheral edema, S_3_ gallop, S_4_ gallop, ascites, hepatomegaly, and jugular vein distension (35.0% missing). However, the results of sensitivity analyses were consistent with the primary analyses, highlighting that the missing data in the PINNACLE Registry were likely missing primarily at random (data not shown).

### Changes in symptoms, signs, and medication prescribing

Changes in HF symptoms and signs (increase or decrease) at clinic visits were infrequently reported (percent of patients): dyspnea appeared in 3.8% and resolved in 2.7%; NYHA class increased in 2.9% and decreased in 2.9%; and number of signs increased in 6.0% and decreased in 5.1%. Changes in HF medication classes were also infrequent: an ACEI/ARB or BB was added in 6.4% and removed in 6.2%; a diuretic was added in 3.7% and removed in 3.8% of patients.

In the 12 months following an index visit, patients had a mean of 0.3 ± 0.63 HF drug class modifications (i.e., drug class addition or removal). Changes in symptoms and signs of HF were infrequently associated with a change in HF medications (Table 2). For example, an ACEI/ARB or BB was added in 15.0% of visits where dyspnea appeared and removed in 18.7% of visits where dyspnea resolved; a diuretic was added in 9.1% of visits where dyspnea appeared and removed in 10.3% of visits where dyspnea resolved. Changes in medication treatment patterns associated with changes in other signs and symptoms followed a similar pattern (Table 2). Rate ratios tended to follow a pattern that indicated an increased frequency of the addition of a medication class associated with symptom or sign worsening and a decreased frequency of the addition of a medication class associated with symptom or sign improvement, compared with no change in symptoms or signs (Table 2).

**Table 2:** Relationship of HF symptoms and signs and treatment patterns by visit pairings

	Treatment Patterns (ACEI, ARB, or BB)^a^					Treatment Patterns (Diuretic)^a^				
	Increase (*n* = 193,516)^b^	No Changes (n = 2,611,047)	Decrease (*n* = 186,627)	Rate Ratio for Treatment Increase	Rate Ratio for Treatment Decrease	No to Yes (*n* = 111,593)	No Changes (*n* = 2,771,748)	Yes to No (*n* = 115,103)	Rate Ratio for Treatment Increase	Rate Ratio for Treatment Decrease
NYHA class change, %^c^										
Increase (*n* = 24,422)	7.8	84.7	7.2	1.3	1.2	4.4	91.0	4.6	1.4	1.2
No changes (*n* = 789,561)	5.9	87.7	6.1	–	–	3.2	92.9	3.8	–	–
Decrease (n = 24,242)	7.5	84.1	7.9	1.3	1.3	5.9	89.7	4.4	1.8	1.2
Dyspnea change, %^c^										
No to yes (*n* = 90,972)	15.0	79.5	5.2	2.5	1.0	9.1	87.9	3.0	2.6	0.9
No changes (n = 2,244,909)	6.0	88.2	5.5	–	–	3.5	93.0	3.5	–	–
Yes to no (*n* = 63,836)	4.4	76.6	18.7	0.7	3.4	2.7	87.0	10.3	0.8	2.9
Orthopnea change, %^c^										
No to yes (*n* = 46,531)	14.4	80.7	4.6	2.3	0.8	8.3	89.0	2.7	2.2	0.7
No change (n = 46,531)	6.3	87.6	5.9	–	–	3.7	92.6	3.7	–	–
Yes to no (*n* = 39,180)	3.8	79.8	16.1	0.6	2.7	2.5	89.2	8.3	0.7	2.2
Changes in signs, %^c,d^										
Increase (*n* = 99,405)	12.9	80.7	6.0	2.1	1.0	8.7	87.8	3.4	2.5	0.9
No changes (n = 1,466,063)	6.1	87.8	5.8	–	–	3.5	92.9	3.6	–	–
Decrease (n = 84,426)	5.4	81.1	13.1	0.9	2.3	3.1	88.8	8.1	0.9	2.3

N = visit pairings; individual patients often represented more than once

*ACEI* angiotensin-converting enzyme inhibitor, *ARB* angiotensin receptor blocker, *BB* beta blocker, *NYHA* New York Heart Association

^a^Treatment patterns were grouped into 1 of 3 categories: ‘increase’ (no to yes), ‘decrease’ (yes to no), or ‘no change’ in number of HF medication classes (ACEI, ARB, BB, and diuretic therapies)

^b^Unit of analysis throughout the table is cardiology outpatient visits

^c^Row percentages do not add up to 100% as a small number of treatment pattern changes that could not be determined were observed

^d^Changes in number of physical signs of HF present out of 7 possible (rales, ascites, peripheral edema, hepatomegaly, third heart sound, fourth heart sound, jugular vein distension)

### Patients with LVEF < 40%

For the 75,107 patients with an LVEF < 40%, baseline demographic and clinical characteristics were similar to the overall study population; however, there was a greater percentage of men in the LVEF < 40% subgroup (69.1% vs 54.9%). Patients with LVEF < 40% and available data were mainly NYHA functional class 2 (46.5%) or 1 (29.0%) and more likely to be prescribed HF medications: BB (85.2%), a diuretic (70.0%), an ACEI (58.6%), or an ARB (18.5%) (Table 1).

Consistent with the results of the overall study population, patients with LVEF < 40% also rarely reported changes in symptoms and signs of HF or in HF medication class, with the majority of patients reporting no changes after the index date (Table 3). The rate ratios for treatment increase or decrease associated with changes in HF symptoms and signs followed a similar pattern as that observed for the overall population (Table 3).

**Table 3:** Patients with LVEF < 40%

	Treatment Patterns (ACEI, ARB, or BB)^a^					Treatment Patterns (Diuretic)^a^				
	Increase (*n* = 36,478)^b^	No changes (*n* = 507,371)	Decrease (*n* = 34,091)	Rate Ratio for Treatment Increase	Rate Ratio for Treatment Decrease	No to Yes (*n* = 22,015)	No changes (*n* = 537,855)	Yes to No (*n* = 19,276)	Rate Ratio for Treatment Increase	Rate Ratio for Treatment Decrease
NYHA class change, %^c^										
Increase (*n* = 8452)	8.1	83.8	7.8	1.5	1.3	6.1	89.8	4.0	1.9	1.1
No changes (*n* = 156,801)	5.5	88.3	6.0	–	–	3.3	93.2	3.5	–	–
Decrease (*n* = 8370)	7.7	85.1	7.1	1.4	1.2	4.5	91.9	3.7	1.4	1.1
Dyspnea change, %^c^										
No to yes (*n* = 20,833)	14.5	80.4	4.8	2.5	0.9	9.2	88.0	2.8	2.6	0.9
No changes (*n* = 435,120)	5.8	88.6	5.4	–	–	3.6	93.3	3.2	–	–
Yes to no (n = 13,026)	4.3	83.8	11.6	0.7	2.2	2.7	92.2	5.1	0.8	1.6
Orthopnea change, %^c^										
No to yes (*n* = 10,720)	13.4	82.0	4.5	2.2	0.8	8.7	88.5	2.8	2.4	0.8
No changes (*n* = 463,734)	6.0	88.1	5.7	–	–	3.7	93.0	3.3	–	–
Yes to no (*n* = 7659)	4.2	87.3	8.4	0.7	1.5	2.8	92.7	4.5	0.8	1.4
Changes in signs, %^c,d^										
Increase (*n* = 24,634)	14.0	79.4	6.3	2.4	1.1	9.6	87.0	3.5	2.7	1.0
No changes (*n* = 275,342)	5.9	88.2	5.7	–	–	3.5	93.1	3.4	–	–
Decrease (n = 18,282)	5.5	85.5	8.6	0.9	1.5	3.3	91.8	4.9	0.9	1.4

N = visit pairings; individual patients often represented more than once

*ACEI* angiotensin-converting enzyme inhibitor, *ARB* angiotensin receptor blocker, *BB* beta blocker, *NYHA* New York Heart Association, *LVEF* left-ventricular ejection fraction

^a^Treatment patterns were grouped into 1 of 3 categories: ‘increase’ (no to yes), ‘decrease’ (yes to no), or ‘no change’ in number of HF medication classes (ACEI, ARB, BB, and diuretic therapies)

^b^Unit of analysis throughout the table is cardiology outpatient visits

^c^Row percentages do not add up to 100% as a small number of treatment pattern changes that could not be determined were observed

^d^Changes in number of physical signs of HF present out of 7 possible (rales, ascites, peripheral edema, hepatomegaly, third heart sound gallop, fourth heart sound gallop, jugular vein distension)

### Patients ≤65 and > 65 years of age

For the 374,580 patients > 65 years of age, with the exception of age (mean [SD]: 77.0 ± 7.6 years), baseline demographic and clinical characteristics were analogous to the overall study population. Changes in the prescribing of HF medication were similarly rarely observed irrespective of age. For patients ≤65 and > 65 years of age at index, the rate ratios for treatment increase or decrease associated with changes in HF symptoms and signs followed a similar pattern as that observed for the overall population (Tables 4 and 5).

**Table 4:** Patients ≤65 years of age

	Treatment Patterns (ACEI, ARB, or BB)^a^					Treatment Patterns (Diuretic)^a^				
	Increase (*n* = 57,100)^b^	No Changes (*n* = 754,371)	Decrease (*n* = 51,608)	Rate Ratio for Treatment Increase	Rate Ratio for Treatment Decrease	No to Yes (*n* = 30,326)	No Changes (*n* = 802,586)	Yes to No (*n* = 32,273)	Rate Ratio for Treatment Increase	Rate Ratio for Treatment Decrease
NYHA class change, %^c^										
Increase (*n* = 6604)	8.2	83.4	8.0	1.3	1.4	5.9	89.8	4.2	2.0	1.1
No changes (*n* = 247.910)	6.2	87.6	5.9	–	–	3.0	93.3	3.7	–	–
Decrease (*n* = 7455)	8.3	84.2	7.2	1.3	1.2	4.5	90.7	4.8	1.5	1.3
Dyspnea change, %^c^										
No to yes (*n* = 25.590)	14.0	80.3	5.3	2.3	1.0	7.8	89.1	3.1	2.4	0.9
No changes *n* = 665,509)	6.2	88.2	5.3	–	–	3.3	93.3	3.4	–	–
Yes to no (*n* = 17,044)	5.0	77.0	17.7	0.8	3.3	3.0	87.5	9.6	0.9	2.8
Orthopnea change, %^c^										
No to yes (*n* = 12,903)	12.3	82.4	4.9	1.9	0.9	6.7	90.4	2.9	1.9	0.8
No changes (*n* = 729,881)	6.5	87.5	5.7	–	–	3.5	92.9	3.7	–	–
Yes to no (*n* = 9829)	4.8	81.1	13.8	0.7	2.4	2.9	90.1	7.0	0.8	1.9
Changes in signs, %^c,d^										
Increase (n = 25,689)	12.8	81.1	5.7	2.0	1.0	7.9	88.7	3.4	2.4	1.0
No changes (*n* = 428,162)	6.4	87.9	5.5	–	–	3.3	93.2	3.5	–	–
Decrease (*n* = 21,623)	6.3	81.0	12.2	1.0	2.2	3.2	89.1	7.7	1.0	2.2

*ACEI* angiotensin-converting enzyme inhibitor, *ARB* angiotensin receptor blocker, *BB* beta blocker, *NYHA* New York Heart Association

^a^Treatment patterns were grouped into one of three categories: ‘increase’ (no to yes), ‘decrease’ (yes to no), or ‘no change’ in number of HF medication classes (ACEI, ARB, BB, and. Diuretic therapies)

^b^Unit of analysis throughout the table is cardiology outpatient visits

^c^Row percentages do not add up to 100% as a small number of treatment pattern changes that could not be determined were observed

^d^Changes in number of physical signs of HF present out of 7 possible (rales, ascites, peripheral edema, hepatomegaly, third heart sound gallop, fourth heart sound gallop, jugular vein distension)

**Table 5:** Patients > 65 years of age

	Treatment Patterns (ACEI, ARB, or BB)^a^					Treatment Patterns (Diuretic)^a^				
	Increase (*n* = 136,416)^b^	No Changes (n = 1,856,676)	Decrease (*n* = 135,019)	Rate Ratio for Treatment Increase	Rate Ratio for Treatment Decrease	No to Yes (*n* = 81,267)	No Changes (n = 1,969,162)	Yes to No (*n* = 82,830)	Rate Ratio for Treatment Increase	Rate Ratio for Treatment Decrease
NYHA class change, %^c^										
Increase (*n* = 17,638)	7.3	84.4	7.9	1.3	1.3	5.9	89.6	4.5	1.7	1.2
No changes (*n* = 541,651)	5.8	87.7	6.2	–	–	3.4	92.7	3.9	–	–
Decrease (*n* = 16,967)	7.6	84.9	7.2	1.3	1.2	4.4	91.1	4.5	1.3	1.2
Dyspnea change, %^c^										
No to yes (*n* = 65,382)	15.3	79.2	5.1	2.6	0.9	9.6	87.5	2.9	2.7	0.8
No changes (n = 1,579,400)	5.9	88.2	5.7	–	–	3.6	92.8	3.5	–	–
Yes to no (n = 46,792)	4.1	76.4	19.1	0.7	3.4	2.5	86.9	10.6	0.7	3.0
Orthopnea change, %^c^										
No to yes (*n* = 33,628)	15.2	80.0	4.5	2.5	0.8	9.0	88.5	2.6	2.4	0.7
No changes (n = 1,706,439)	6.2	87.6	6.0	–	–	3.8	92.5	3.8	–	–
Yes to no (*n* = 29,351)	3.5	79.4	16.8	0.6	2.8	2.4	88.9	8.8	0.6	2.3
Changes in signs, %^c,d^										
Increase (*n* = 72,716)	13.0	80.5	6.1	2.1	1.0	9.0	87.5	3.5	2.6	1.0
No changes (n = 1,037,901)	6.1	87.8	5.9	–	–	3.5	92.8	3.7	–	–
Decrease (*n* = 62,803)	5.1	81.1	13.5	0.8	2.3	3.1	88.6	8.3	0.9	2.2

N = visit pairings; individual patients often represented more than once

*ACEI* angiotensin-converting enzyme inhibitor, *ARB* angiotensin receptor blocker, *BB* beta blocker, *NYHA* New York Heart Association

^a^Treatment patterns were grouped into 1 of 3 categories: ‘increase’ (no to yes), ‘decrease’ (yes to no), or ‘no change’ in number of HF medication classes (ACEI, ARB, BB, and diuretic therapies)

^b^Unit of analysis throughout the table is cardiology outpatient visits

^c^Row percentages do not add up to 100% as a small number of treatment pattern changes that could not be determined were observed

^d^Changes in number of physical signs of HF present out of 7 possible (rales, ascites, peripheral edema, hepatomegaly, third heart sound gallop, fourth heart sound gallop, jugular vein distension)

## Discussion

For patients with HF, the frequency of cardiology clinic visits, changes in symptoms and signs between visits, changes in HF medication class prescribing patterns at visits, and the relationship between them has not been previously described across the U.S. ambulatory cardiology setting. The current study found that the majority of patients with HF had 2 or more cardiology clinic visits in a year. Health records from these visits rarely recorded changes in dyspnea, orthopnea, NYHA functional class, or physical signs between visits. Further, HF medication class modifications were infrequent. Although predictable associations between changes in symptoms/signs and drug therapy were observed, particularly the addition of medication with increases in dyspnea and vice versa, the absolute rate of initiation and discontinuation of drug classes was small. This apparent lack of major HF treatment modification during ambulatory visits (recognizing the limitations of health record data generally and the absence of medication dosing here specifically) is a finding that warrants further investigation, either as a potential opportunity to more proactively optimize HF medication prescribing at cardiology visits (particularly in the HF population with reduced LVEF) or as an opportunity to reduce some routine follow-up visits in the absence of clinical change.

The vast majority of real-world HF data have been collected from inpatient settings, including the hospital-based registries such as the Organized Program to Initiate Lifesaving Treatment in Hospitalized Patients with Heart Failure (OPTIMIZE-HF) [11] and Get With The Guidelines®-Heart Failure (GWTG-HF) [14]. Ambulatory data have primarily been collected from randomized controlled trials, with limitations in external validity due to narrow inclusion criteria and algorithms for medication management [18, 22, 23]. A real-world ambulatory evaluation of care for patients with HF was provided through Improve the Use of Evidence-Based Heart Failure Therapies in the Outpatient Setting (IMPROVE-HF) [12, 19, 24], but those data represented a narrow cohort of participating centers and are becoming increasingly dated. PINNACLE represents the largest ambulatory data available for Americans diagnosed with a variety of CV conditions (coronary artery disease, hypertension, HF, and atrial fibrillation) from outpatient practices across the U.S. [19]. Here, the PINNACLE Registry has provided preliminary insights into what occurs at ambulatory cardiology visits for patients with HF.

Adherence to treatment guidelines has been shown to improve outcomes of patients with HF, including health-related quality of life. Poor health-related quality of life has been associated with many factors including greater symptom burden in patients with HF [7]. A patient-centered focus on symptom deterioration may facilitate more rapid and adequate care and reduce the need for hospitalizations [8, 25]. In the current study, a lack of change in the index HF treatment was most often observed in the presence of worsening symptoms and signs. Previous studies have reported various rationales for not modifying treatment patterns in accordance with HF treatment guidelines, including medical reasons (e.g., tolerability) and human reasons (e.g., clinical inertia, patient preferences) [26]. Studies have also found lack of adherence to HF guidelines may more often be seen in older patients or those with comorbidities, due to concerns with clinical complexity of treatment and limited potential benefits for these patients [27]. Because of the nature of PINNACLE data, we are unable to comment on the reasons here, other than to say that these patterns were seen across LVEF and age groups. Future research could examine the association of dosage regimen optimization with symptom control to inform clinical practice.

A number of limitations with regard to the current study should be considered. Because indications for BB, ACEI, ARB, aldosterone antagonists, and newer agents are all dependent on LVEF, the lack of LVEF data in nearly half of patients limits the analysis. Fortunately, with the large sample size in PINNACLE, we were able to look at the subgroup of patients with HF with reduced LVEF and found that medication changes in this group were similarly low. The loss of detailed medication information when data were transformed for analysis (i.e., type of diuretic and dosage information) significantly limited the ability to understand the scope of therapy adjustments occurring at clinic visits [18, 28, 29]. For example, a patient could have her furosemide dose increased and spironolactone added, but these medication changes would still be classified by PINNACLE as “unchanged” diuretic. PINNACLE data capture does not allow for an analysis of HF medication dosing. Continuous advances in the interface and processing of electronic health records within PINNACLE are ongoing and should improve medication data capture in the future. Finally, outpatient cardiology practices voluntarily participating in the PINNACLE Registry may not optimally represent practice patterns across the U.S.; however, the characteristics of practices and patients in PINNACLE have been shown to mirror the broader U.S. cardiology ambulatory population.

## Conclusions

Serial ambulatory HF visits in U.S. cardiology practices were common and rarely involved changes in symptoms and signs. HF medications were seldom added or removed at a class level. These findings may suggest opportunities for more proactive medication class optimization or, alternatively, for more symptom-based visit scheduling.

## Abbreviations

ACEI: Angiotensin-converting enzyme inhibitors
ARB: Angiotensin receptor blockers
BB: Beta blockers
CV: Cardiovascular
HF: Heart failure
LVEF: Reduced left-ventricular ejection fraction
NCDR: National cardiovascular data registry
NYHA: New York heart association
PINNACLE: Practice innovation and clinical excellence
S_3_ gallop: Third heart sound
S_4_ gallop: Fourth heart sound
